# Comparative population structure of two dominant species, *Shinkaia crosnieri* (Munidopsidae: *Shinkaia*) and *Bathymodiolus platifrons* (Mytilidae: *Bathymodiolus*), inhabiting both deep‐sea vent and cold seep inferred from mitochondrial multi‐genes

**DOI:** 10.1002/ece3.2132

**Published:** 2016-04-23

**Authors:** Yanjun Shen, Qi Kou, Weitao Chen, Shunping He, Mei Yang, Xinzheng Li, Xiaoni Gan

**Affiliations:** ^1^ The Key Laboratory of Aquatic Biodiversity and Conservation of the Chinese Academy of Sciences Institute of Hydrobiology Chinese Academy of Sciences Wuhan 430072 Hubei China; ^2^ University of the Chinese Academy of Sciences Beijing 100039 China; ^3^ Institute of Oceanology Chinese Academy of Sciences Qingdao 266071 China

**Keywords:** Chemosynthetic fauna, environmental heterogeneity, genetic diversity, genetic isolation, population distribution patterns

## Abstract

Deep‐sea hydrothermal vents and cold seeps, limited environments without sunlight, are two types of extreme habitat for marine organisms. The differences between vents and cold seeps may facilitate genetic isolation and produce population heterogeneity. However, information on such chemosynthetic fauna taxa is rare, especially regarding the population diversity of species inhabiting both vents and cold seeps. In this study, three mitochondrial DNA fragments (the cytochrome *c* oxidase submit I (COI), cytochrome *b* gene (Cytb), and 16S) were concatenated as a mitochondrial concatenated dataset (MCD) to examine the genetic diversity, population structure, and demographic history of *Shinkaia crosnieri* and *Bathymodiolus platifrons*. The genetic diversity differences between vent and seep populations were statistically significant for *S. crosnieri* but not for *B. platifrons*. *S. crosnieri* showed less gene flow and higher levels of genetic differentiation between the vent and seep populations than *B. platifrons*. In addition, the results suggest that all the *B. platifrons* populations, but only the *S. crosnieri* vent populations, passed through a recent expansion or bottleneck. Therefore, different population distribution patterns for the two dominant species were detected; a pattern of population differentiation for *S. crosnieri* and a homogeneity pattern for *B. platifrons*. These different population distribution patterns were related to both extrinsic restrictive factors and intrinsic factors. Based on the fact that the two species were collected in almost identical or adjacent sampling sites, we speculated that the primary factors underlying the differences in the population distribution patterns were intrinsic. The historical demographics, dispersal ability, and the tolerance level of environmental heterogeneity are most likely responsible for the different distribution patterns.

## Introduction

Deep‐sea hydrothermal vents, first discovered on the Galapagos Rift in the eastern Pacific Ocean in 1977 (Corliss et al. 1979), are typically located in mid‐ocean ridges and back‐arc spreading centers. Vents have extremely high temperatures, high pressure levels, and high levels of toxins (Yang et al. 2013). Cold seeps, situated in subduction zones with ecosystems similar to vents, are areas where chemically modified fluids derived from hydrocarbon reservoirs, methane hydrates, pore waters in sediments, and sites of organic enrichment (such as whale skeletons) are released into the ocean (Van Dover et al. 2002). A few decades ago, sunlight was considered the essential element for life because photosynthesis provides primary production for the biosphere. However, numerous unique “chemosynthetic fauna”, which depend primarily on energy supplied by the chemosynthesis of bacterial endosymbionts, are found in the limited environments without sunlight (Tirmizi and Javed 1980; Kenk and Wilson 1985; Hessler and Lonsdale 1991; Kikuchi and Ohta 1995; Watsuji et al. 2010). Knowledge regarding the biological communities around vents and cold seeps led to a profound change in our perception of deep‐sea life (Van Dover et al. 2002).

Within deep‐sea chemosynthetic ecosystems, particularly hydrothermal vents, studies of population structure and gene flow has recently received much attention for many species, such as giant tubeworms (Hurtado et al. 2004; Coykendall et al. 2011), mussels (Won et al. 2003), swarming shrimp (Teixeira et al. 2012), and provannid gastropods (Kojima et al. 2001; Teixeira et al. 2012). Population diversity studies are also reported for deep‐sea chemosynthetic communities at the methane seeps in the Gulf of Mexico and eastern Atlantic (Carney et al. 2006; Olu et al. 2010). From these studies, we are beginning to understand the extent of genetic connectivity among deep‐sea populations associated with chemosynthetic ecosystems. However, few studies have been conducted to examine the genetic diversity and population structure for species inhabiting both vents and cold seeps, especially for the dominant species. As we know, relying on chemoautotrophic bacteria for nutritional support, all known species of macroorganisms in hydrothermal vents and cold seeps are highly specialized for a symbiotic lifestyle (Cavanaugh 1983; Fialamedioni and Lepennec 1988; Fisher 1990). The differences between the ecosystems of vents and cold seeps may facilitate the genetic isolation of species in both environments. Therefore, it is particularly important to determine the extent of population differentiation and genetic isolation for these species.

Bathymodiolus (Mytilidae: *Bathymodiolus*) mussels, which are the dominant species in vents and cold seeps, have been described beyond 18 species. Only three Bathymodiolus species in Japanese waters (*B. japonicus*,* B. platifrons*, and *B. aduloides*) are capable of inhabiting both vents and cold seeps. In particular, the same haplotypes have been found to be shared by *B. platifrons* from distant localities and highly differentiated environments (Miyazaki et al. 2004). *Shinkaia crosnieri* (Munidopsidae: *Shinkaia*) is also considered the dominant member of the fauna inhabiting many deep‐sea hydrothermal vents and minority cold seeps, including Hatoma Knoll, the Formosa Ridge off southwest Taiwan, and the Okinawa Trough (Chevaldonne and Olu 1996; Tsuchida and Fujikura 2000; Watabe and Miyake 2000; Martin and Haney 2005; Tsuchida et al. 2007). Previous studies of *B. platifrons* from two seeps and one vent revealed no genetic differentiation between the mussel populations of either species from cold seeps versus vents and suggested high adaptability to deep‐sea chemosynthetic environments (Miyazaki et al. 2004). However, the distribution and population structure of *S. crosnieri* have been reported only at hydrothermal vents in the southern Okinawa Trough (Tsuchida et al. 2003). The two dominant species (*B. platifrons* and *S. crosnieri*) are ideal models as they exhibit various population distances and inhabit different habitat types.

In this study, three mitochondrial DNA fragments (the cytochrome *c* oxidase submit I (COI), cytochrome *b* gene (Cytb), and 16S) were employed as a mitochondrial concatenated dataset (MCD) to evaluate the genetic diversity, genetic structure, and demographic history of *B. platifrons* and *S. crosnieri*. The current study also sought to explore the adaptability of the two dominant species to different deep‐sea chemosynthetic environments.

## Materials and Methods

The experiments were performed in accordance with the recommendations of the Ethics Committee of the Institute of Hydrobiology, Chinese Academy of Sciences. The policies were enacted according to the Chinese Association for Laboratory Animal Sciences and the Institutional Animal Care and Use Committee (IACUC) protocols.

### Sampling, identification, and DNA extraction

**Figure 1:**
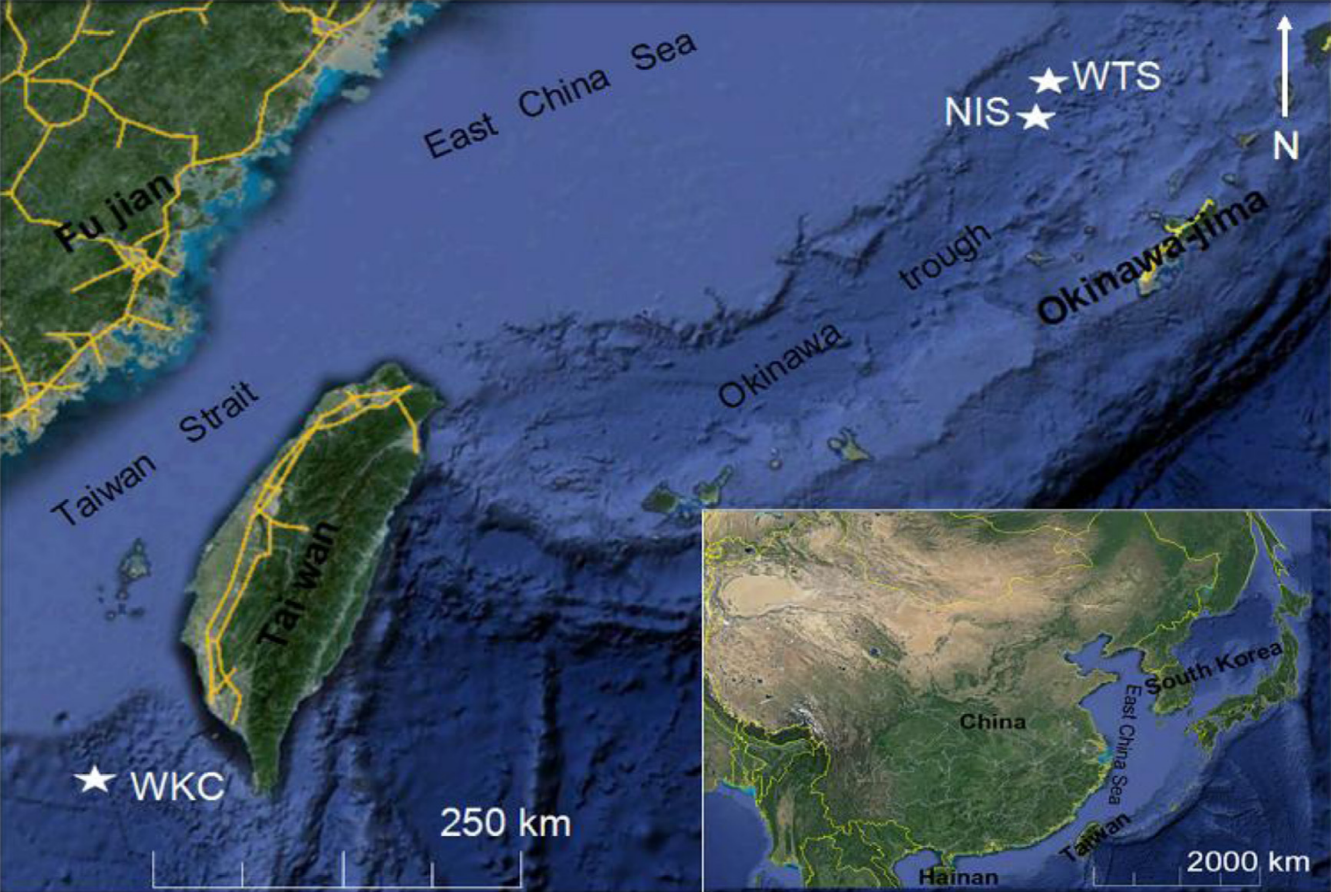
Sampling locations in the Okinawa Trough and Taiwan Strait near the South China Sea. WKC is a cold seep site; NIS and WTS are vent sites.

A total of 64 specimens of *S. crosnieri* from three vents and one cold seep habitat, and 52 specimens of *B. platifrons* from two vents and one cold seep habitat were collected by the manned submersible “Jiaolong” during June 2013 and the ROV “Faxian” during April 2014. All the sample information and photographs used in this study are provided in Table 1 and the sampling sites are mapped in Fig. 1. All the specimens were frozen and preserved at −80°C or in 100% ethanol and deposited in the Institute of Oceanology, Chinese Academy of Science.

**Table 1:** Information about the samples used in this study

Species	Sampling site	Depth (m)	Location	Habitat type	Population name	*N*	Date
*Shinkaia crosnieri*	West of Tori‐shima	996.9	27°47.441754′N 126°53.8029781′E	Vent	WTS1	44	2014.4.17
	Northwest of Iheya‐shima	1361.2	27°33.06928′N 126°58.13082′E	Vent	NIS	6	2014.4.20
	West of Tori‐shima	983.5	27°47.43526′N 126°58.82327′E	Vent	WTS2	5	2014.4.22
	West of Kaohsiung City	1119	22°6.915′N 119°17.123′E	Seep	WKC	9	2013.6.18
*Bathymodiolus platifrons*	Northwest of Iheya‐shima	1361.2	27°33.06928′N 126°58.13082′E	Vent	NIS1	13	2014.4.20
	Northwest of Iheya‐shima	1390.7	27°33.00178′N 126°58.19623′E	Vent	NIS2	9	2014.4.20
	West of Kaohsiung City	1132	22°6.911′N 119°17.158′E	Seep	WKC	30	2013.6.17

Sampling site: location roughly based on longitude and latitude; Population name: from initial of sampling site; *N*: number of specimens.

The distance: WTS1‐NIS: 27.6 km, WKC‐NIS: 983.8 km, WKC‐WTS1: 999.8 km, WTS1‐WTS2: <0.1 km, NIS1‐WKC: 983.7 km, NIS2‐WKC: 983.6 km, NIS1‐NIS2: 0.2 km.

The species‐level morphological identification was performed based on the original morphological descriptions, locality data, and additional information of *S. crosnieri* (Baba and Williams 1998; Chan et al. 2000) and of *B. platifrons* (Hashimoto and Okutani 1994; Barry et al. 2002).

The total genomic DNA of *S. crosnieri* was extracted from a small piece of abdominal muscle tissue using the E.Z.N.A.^®^ Tissue DNA Kit (OMEGA, Wuhan, China) following the manufacturer's instructions. Several bivalve species have an unusual mode of mtDNA inheritance known as doubly uniparental inheritance (DUI) (Zouros et al. 1994a; Zouros et al. 1994b; Garrido‐Ramos et al. 1998; Plazzi and Passamonti 2010). DUI species have two highly differentiated mitochondrial genomes, one of which follows the standard mode of maternal inheritance (known as type F) and the other is transmitted through sperm and found almost entirely in the male gonad (known as type M) (Mizi et al. 2005). Phylogenetic and population analyses require comparisons between orthologous sequences and M‐or F‐type genes under DUI that are not orthologous sequences (Plazzi and Passamonti 2010). Therefore, the total genomic DNA of *B. platifrons* was obtained from a small section of adductor or foot muscle, which carries very little M‐type mtDNA in DUI species (Grant and Bowen 1998). Then, the extracted DNA was suspended in distilled water and stored at −20°C.

### PCR amplification and sequencing

Three mitochondrial DNA fragments, COI, Cytb, and 16S were amplified using the primers listed in Table S1 (Palumbi et al. 1991; Folmer et al. 1994; Merritt et al. 1998; Baco‐Taylor 2002; Passamonti 2007; Radulovici et al. 2009). The final reaction volume of 30 μL contained 21.125 μL of sterilized ultrapure water, 3.0 μL of 10**×** PCR buffer (including MgCl_2_), 1.5 μL of each primer (10 mmol/L), 1.5 μL of dNTPs (2.5 mmol/L each), 0.375 μL of Taq DNA polymerase (2.5 U/μL, TaKaRa Bio, Shanghai, China), and 1.0 μL of DNA template (50–100 ng/μL). The cycling parameters were 94°C for 5 min; 32 cycles of 94°C for 40 sec, 43–48°C for 30 sec and 72°C for 1 min, and a final elongation step at 72°C for 10 min. The PCR products were visualized on 1.2% low‐melting agarose gels stained with ethidium bromide. Then, the products were purified and sequenced on an ABI3730 XL sequencing system using the primers described in Table S1.

### Molecular data analysis

The sequences obtained in both directions were checked by the sequence peak figure and then assembled based on the contigs using the DNASTAR Lasergene package (DNASTAR, Inc., Madison, WI, USA). The sequences were aligned and trimmed to the same length using the software package MEGA 5.0 (Tamura et al. 2011). Then, the MCD was used directly for the subsequent population analyses.

The average genetic distances within and between populations were estimated according to the Kimura 2‐parameter (Kimura 1980) model in MEGA 5.0 (Tamura et al. 2011). Genetic diversity is reflected in the measures of nucleotide diversity (*π*) and haplotype diversity (*h*) (Nei 1987), and the values for each population were calculated using DnaSP 5.10 (Librado and Rozas 2009). The maximum likelihoods (ML) for phylogenetic analyses were assembled in PhyML 3.0 (Guindon and Gascuel 2003) with 1000 replicates, and the most appropriate model of DNA substitution, which is TIM2+I+G for *S. crosnieri* and HKY+I for *B. platifrons*, was identified by ModelTest 3.7 (Posada and Crandall 1998). *Alvinocaris longirostris* (GenBank: AB821296) and *Geothelphusa dehaani* (GenBank: AB187570) were set as outgroups for *S. crosnieri*. *Mytilus californianus* (GenBank: NC_015993) and *Mytilus edulis* (GenBank: NC_006161) were set as outgroups for *B. platifrons*.

Median‐joining network (MJN) analysis was performed with Network 4.6 (Bandelt et al. 1999) to depict the relationships among all the haplotypes. The pairwise genetic divergences between the populations were estimated using *F*‐statistics (*F*_ST_) with 10,000 permutations, based on the distance method in Arlequin 3.5 (Excoffier and Lischer 2010). Analysis of the molecular variance analysis (AMOVA) was used to analyze the hierarchal population structure in Arlequin.

The population demography (e.g., bottlenecks or expansions) for the vent and seep populations were examined using two different approaches. First, the demographic history was investigated by comparing the mismatch distributions in each habitat‐type sample with those expected in stationary and expanding populations using DnaSP 5.10 (Librado and Rozas 2009). In addition, we tested the goodness‐of‐fit of the actual distributions with the expected distributions using a model of population expansion by calculating the Harpending's raggedness index (*r*) (Harpending 1994) and by assessing significance with 1000 permutations. Second, Tajima's D (Tajima 1989) was also applied to seek evidence of demographical expansions in Arlequin 3.5 (Excoffier and Lischer 2010). In addition, gene flow (*N*_m_) was evaluated from GammaSt (Nei 1982), and *F*_ST_ (Slatkin 1985; Hudson et al. 1992) was evaluated using DnaSP 5.10 (Librado and Rozas 2009). The geographic distances between populations were estimated using Google Earth 4.3 based on the longitude and latitude.

## Results

### Sequence information

All three mitochondrial gene fragments were successfully amplified for 64 *S. crosnieri* specimens and 52 *B. platifrons* specimens. Meanwhile, COI (GenBank accession nos. KR003111‐KR003157), Cytb (GenBank accession nos. KR003178‐KR003222), and 16S (GenBank accession nos. KR003236‐KR003244) of *S. crosnieri* and COI (GenBank accession nos. KR003158‐KR003177), Cytb (GenBank accession nos. KR003223‐KR003235), and 16S (GenBank accession nos. KR003245‐KR003280) of *B*. *platifrons* were deposited in GenBank. For *S. crosnieri*, the MCD sequences (1480 bp) contained 111 variable sites, 65 of which were parsimony informative. For *B. platifrons*, MCD sequences (1539 bp) contained 45 variable sites, 11 of which were parsimony informative.

### *S. crosnieri*: genetic diversity and population structure

The number of haplotypes (H), the haplotype diversity (*h*), and nucleotide diversity (*π*) for each population of *S. crosnieri* are presented in Table 2. All populations showed high haplotype diversity and there was no shared haplotypes except one each for the vent and cold seep populations. All populations also showed high haplotype diversity, and several shared haplotypes were detected among the vent and cold seep populations. A comparison of the genetic diversity showed that the haplotype and nucleotide diversities were lowest in the cold seep population (*h = *0.972, *π *= 0.0062) and highest in the vent WTS1 population (*π *= 0.0062).

**Table 2:** DNA polymorphism, neutrality tests and mismatch distribution values for all *Shinkaia crosnieri* and *Bathymodiolus platifrons* populations

Species	Population	*n*	*H*	*h*	*π*	Tajima's D	Hri	Tau
*Shinkaia crosnieri*	WTS1	44	44	1.000	0.0062			
	NIS	6	6	1.000	0.0039			
	WTS2	5	5	1.000	0.0057			
	Whole vents dataset	55	54	0.999	0.0059	−1.824**	0.005	8.2
	WKC	9	8	0.972	0.0032	−0.745	0.072	2.9
	Whole dataset	64	62	0.999	0.0110	−1.057	0.004	6.9
*Bathymodiolus platifrons*	NIS1	13	11	0.974	0.0018			
	NIS2	9	7	0.917	0.0020			
	Whole vents dataset	22	17	0.965	0.0020	−1.958*	0.032	3.1
	WKC	30	22	0.968	0.0021	−1.919*	0.030	3.1
	Whole dataset	52	36	0.970	0.0021	−2.298***	0.027	2.8

*n*: sample size; *H*: number of haplotypes; *h*: haplotype (gene) diversity; *π*: nucleotide diversity; Tau: time since expansion, expressed in units of mutational time; Hri: Harpending's raggedness index; Significant: * = *P* < 0.05; ** = *P* < 0.01; *** = *P* < 0.001.

Based on the Kimura 2‐parameter model, the overall average genetic distance among individuals was 0.0112 for *S. crosnieri*. The mean genetic distances within and between each of the populations are shown in Table 3. The overall mean intrapopulation genetic distance of the WTS1 population (0.0062) was the largest among the four populations and was smallest for the WKC population (0.0032). The genetic distances among WTS1, WTS2, and NIS were very small, but the genetic distances between WKC and all the vent populations were higher.

**Table 3:** Population pairwise *F*_ST_ fixation index (below the diagonal), mean genetic distances within (on the diagonal) and between (above the diagonal) populations of *Shinkaia crosnieri* and *Bathymodiolus platifrons*

Species	Population	WTS2	WTS1	NIS	WKC
*Shinkaia crosnieri*	WTS2	0.0057	0.0061	0.0057	0.0272
	WTS1	0.0228	0.0062	0.0051	0.0276
	NIS	0.1699**	−0.0191	0.0039	0.0275
	WKC	0.8476***	0.7954***	0.8718***	0.0032
*Bathymodiolus platifrons*	Population	NIS1	NIS2	WKC	
	NIS1	0.0018	0.0021	0.0020	
	NIS2	0.0341	0.0020	0.0025	
	WKC	0.0440	0.1718***	0.0021	

Significant: *=*P* < 0.05; ** =*P* < 0.01; *** =*P* < 0.001.

**Figure 2:**
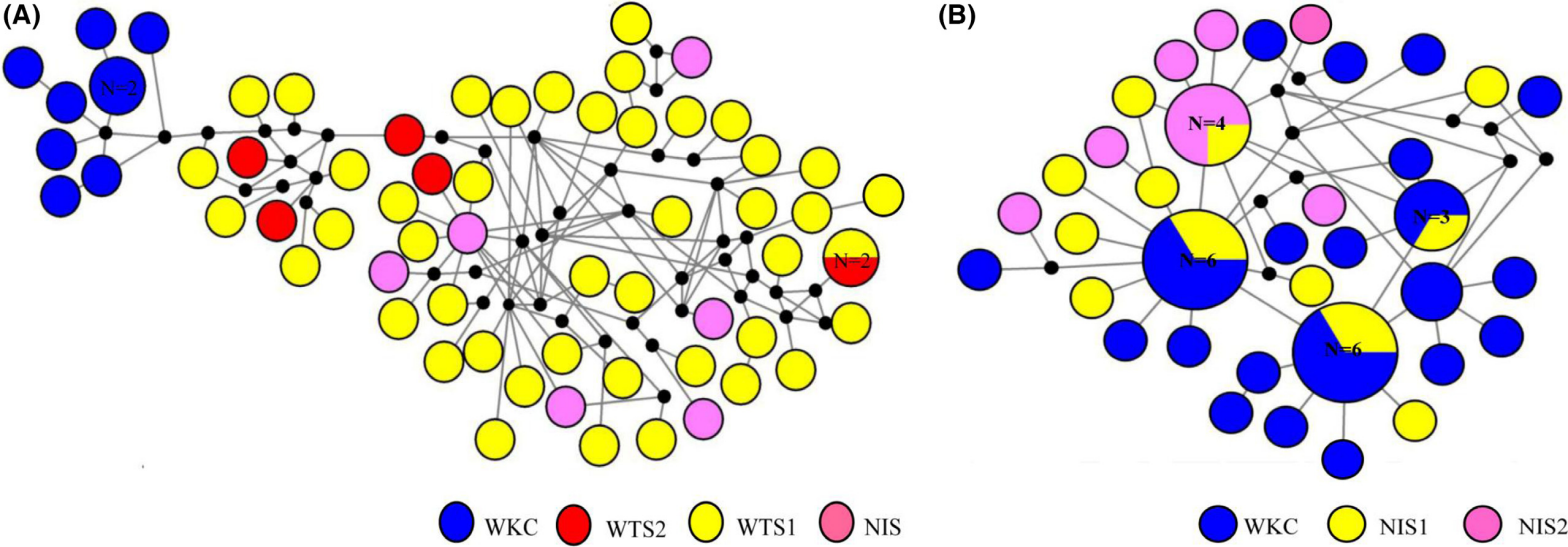
Median‐joining network of haplotypes of *S. crosnieri* and *B. platifrons*. Each circle represents a haplotype, and its diameter is proportional to the frequency. *N* is the number of individuals, and each color shows a different species population. A for *S. crosnieri* and B for *B. platifrons*.

The median‐joining network analysis had a weblike topology, with many singletons connected through multiple nodes, indicating high genetic variability for *S. crosnieri* (Fig. 2A). It was found that haplotypes from the vent and seep populations could be separated and did not share common haplotypes. Moreover, two obvious clades (Vent and Cold Seep) were also identified from the ML tree (Fig. 4A).

For population structure analysis, all samples were divided into vent and seep groups. An AMOVA analysis revealed that most of the molecular variance of *S. crosnieri* occurred among groups (79.70%), whereas the variance among the intragroup populations (0.35%) and within populations (19.95%) was relatively small (*F*_ST_ = 0.801, *P* < 0.001), indicating a high level of geographical population structure (Table 4). In addition, all pairwise *F*_ST_ comparisons between the vent and seep populations were statistically significant (*P* < 0.001). The values of Nm based GammaSt (from 1.08 to 29.93) and *F*_ST_ (from 0.27 to 43.98) indicated the fluctuation of gene flow levels among the four *S. crosnieri* populations (Table S2). The level of gene flow between the vent and seep populations was very limited, which was consistent with the statistically significant *F*_ST_ values among them.

**Table 4:** Analysis of molecular variance for *Shinkaia crosnieri* and *Bathymodiolus platifrons* populations

Species	Source of variation	Variation components	Percentage of variation	*F*_ST_	*P*
*Shinkaia crosnieri*	Among groups	16.3719	79.70	0.801	0.0000
	Among populations within groups	0.0719	0.35		
	Within populations	4.0982	19.95		
*Bathymodiolus platifrons*	Among groups	0.0510	3.00	0.103	0.0001
	Among populations within groups	0.1252	7.35		
	Within populations	1.5268	89.65		

All samples were divided into vent and seep groups. For *S. crosnieri*, the vent group included the WTS1, WTS2, NIS populations, and the seep group included the WKC population. For *B. platifrons*, the vent group included NIS1, NIS2 populations, and the seep group included the WKC population.

**Figure 3:**
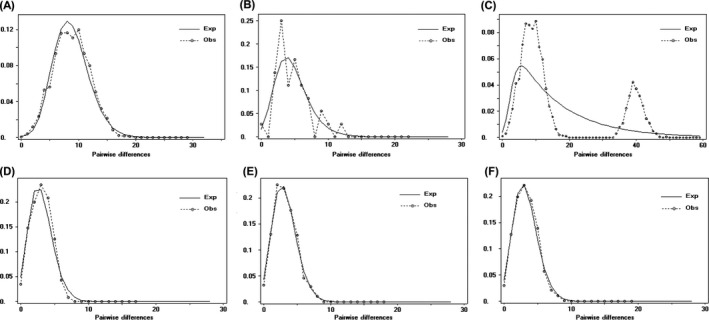
Expected (solid lines) and observed (broken lines) mismatch distribution. For *S. crosnieri,* (A) whole vents dataset; (B) WKC; (C) whole dataset. For *B. platifrons,* (D) whole vents dataset; (E) WKC; (F) whole dataset. *X*‐axis = pairwise differences and *Y*‐axis = frequency.

The shape of the mismatch distribution of *S. crosnieri* was approximately unimodal for the whole vent dataset (Fig. 3A). Nevertheless, the distribution was obviously ragged and multimodal for the seep WKC population (Fig. 3B) and the whole dataset (Fig. 3C). In addition, none of the values for Harpending's raggedness index were significant, and the Tajima's *D* values were statistically significant for only the whole vents dataset (*P* < 0.01) (Table 2).

### *B. platifrons*: genetic diversity and population structure

The values of *H*,* h*, and *π* for each population of *B*. *platifrons* are presented in Table 2. All populations of *B. platifrons* showed high haplotype diversity and several shared haplotypes were detected among the vent and cold seep populations. A comparison of the genetic diversity of *B. platifrons* detected a similar level of haplotype and nucleotide diversities among all populations.

The overall average genetic distance among individuals was 0.0021 for *B. platifrons*. The mean genetic distances within and between each of the populations are shown in Table 3. The overall mean intrapopulation genetic distance of the WKC population (0.0021) was the largest among the three populations, and that of NIS1 (0.0018) was the smallest. Therefore, the genetic distances among all populations were very small.

**Figure 4:**
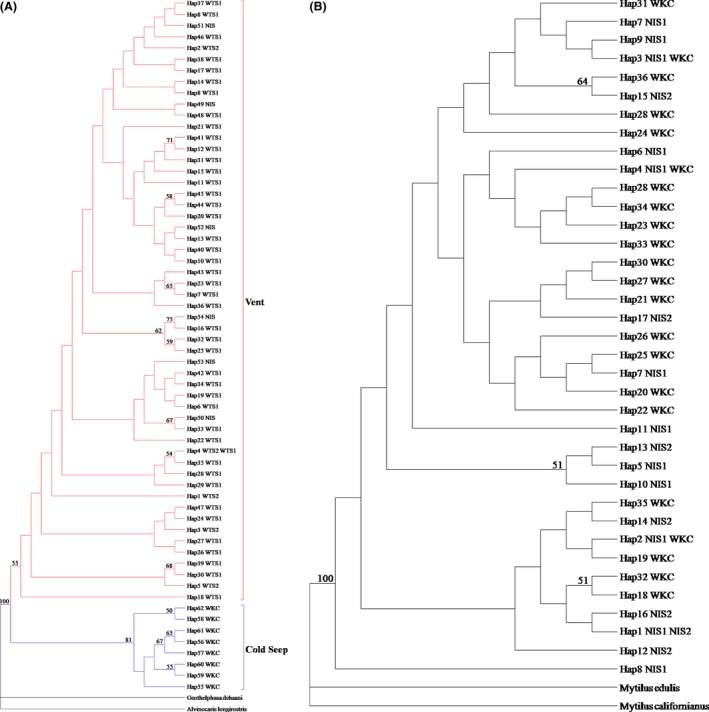
Maximum likelihood tree for *S. crosnieri* and *B. platifrons* based on haplotypes. Vent = all individuals of the vent populations; Cold Seep = all individuals of the seep population. A for *S. crosnieri* and B for *B. platifrons*. Bootstrap values >50 are reported.

The median‐joining network analysis had only several singletons connected through multiple nodes for *B. platifrons* (Fig. 2B). We found that individuals of *B. platifrons* from the vent and seep populations shared haplotypes. In particular, three common haplotypes were shared by the seep WKC and vent NIS1 populations. One common haplotype was shared by the vent NIS1 and NIS2 populations. In addition, the ML tree also showed that some individuals from the vent and seep populations occupied the same clades (Fig. 4B).

For population‐structure analysis, all samples were divided into vent and seep groups. An AMOVA analysis revealed that most of the molecular variance of *B. platifrons* occurred within populations (89.65%), whereas variance among populations within groups (7.35%) and among groups (3.00%) was relatively small (3.00%) (*F*_ST_ = 0.103, *P* < 0.001), indicating a low level of geographical structure (Table 4). In addition, significant *F*_ST_ was found only between WKC and the vent NIS2 population (*P* < 0.001) (Table 3). The values of Nm‐based GammaSt (from 4.78 to 11.17) and *F*_ST_ (from 2.37 to 9.96) indicated a slight stabilization of the gene flow levels among the three *B. platifrons* populations (Table S2). The gene flow levels among the vent populations were obviously large. Especially for vent NIS2 and the seep WKC populations of *B. platifrons*, the gene flow was rather weak, which was also consistent with the statistically significant *F*_ST_ values between them.

All the mismatch distribution shape of *B. platifrons* was obviously unimodal (Fig. 3D, E, F). In addition, none of the values for Harpending's raggedness index was significant, and all the Tajima's *D* values were statistically significant (*P* < 0.01) (Table 2).

## Discussion

### Comparative genetic diversity and population structure

Based on the haplotype and nucleotide diversities, all populations of *S. crosnieri* and *B. platifrons* showed high haplotype diversities but low nucleotide diversities, which are normally associated with an expansion model (Grant and Bowen 1998). All the vent populations showed higher genetic variability than the seep population for *S. crosnieri*, whereas a similar level of the genetic variability was detected among *B. platifrons* populations.

The AMOVA analysis and pairwise *F*_ST_ values indicated that high levels of population structure and genetic differentiation occurred between the vent and seep populations for *S. crosnieri*, but low levels of population structure and genetic differentiation occurred among the *B. platifrons* populations (although the distance between vent and seep are above 983.8 km), except NIS2‐WKC, which was probably related to small sample size. For *S. crosnieri*, the results were also supported by a weblike statistical haplotype network and ML tree analyses. The lack of interpopulation contact might be related to its ecological habits (vent and seep) and the long distance of the distribution areas (the distance between the vent and seep are more than 983.8 km). This phenomenon could be proven by the lack of gene flow between vent and seep populations but higher gene flow levels among vent populations, especially for WTS1‐WTS2. However, for *B. platifrons*, the statistical haplotypes network and ML tree analysis showed that the haplotype distribution did not mirror the geographical origin of the populations because several haplotypes were shared among individuals from different habitat populations. In addition, gene flow was also detected among the populations. The results suggested that extrinsic restrictive factors for the distributions were not related to environmental types (vents vs. seeps) (Olu et al. 1996; Fujikura et al. 2000).

The shape of the mismatch distribution of pairwise differences was ragged or multimodal for the populations at stationary demographic equilibrium, but it is typically smooth or unimodal for populations that have passed through a recent expansion or bottleneck (Rogers and Harpending 1992; Schneider and Excoffier 1999). The smooth and approximately unimodal shape of the mismatch distribution for the whole vent dataset of *S. crosnieri* populations showed that vent populations are likely to have passed through a recent expansion or bottleneck, but cold seep WKC *S. crosnieri* populations did not because of the multimodal shape of the mismatch distribution. The results demonstrated that *S. crosnieri* populations had different demographic histories in vent and cold seep environments. Significant *D* values may be due to factors such as population expansion and bottleneck (ArisBrosou and Excoffier 1996). The significant negative *D* values for the entire vents dataset also could support the results. Nevertheless, for *B. platifrons*, both the obviously unimodal shape and the statistically significant negative D values showed that all populations probably passed through a recent expansion or bottleneck.

Generally, species living in patchy, fragmented, ephemeral habitats are expected to harbor less genetic diversity (Tunnicliffe and Juniper 1990) and are likely to have encountered an expansion or bottleneck. Moreover, cold seeps are relatively stable, whereas some vents persist for only a few decades (Tunnicliffe and Juniper 1990). In this study, the *S. crosnieri* vent populations have passed through an expansion or bottleneck but the cold seep populations have not, indicating that the vent habitats were more fluctuant than the cold seep habitats.

A previous study for Bathymodiolus mussels has also demonstrated extensive intraspecific genetic exchanges between vent and seep sites across thousands of kilometers (Van Dover et al. 2002; Miyazaki et al. 2004; Iwasaki et al. 2006). Our analysis also indicated that genetic exchanges occurred among three populations and was largest between the seep WKC and vent NIS1 populations. Based on these results, a homogeneity pattern was fit for the *B. platifrons* populations. The absence of differentiation between the seep and vent mussels demonstrated that the primary intrinsic factor can most likely be ascribed to the different physiological tolerance of the mussels to pressure (Olu et al. 1996; Fujikura et al. 2000).

### Comparison of different population distribution patterns

In our analyses, different population distribution patterns for the two dominant species were detected. However, the different population patterns were related to extrinsic restrictive factors, such as environmental heterogeneity, geological histories, and oceanic currents, as well as intrinsic factors, such as historical demographics, dispersal ability, and physiology (Iwasaki et al. 2006). The extrinsic restrictive factors for the two species were almost identical because the sampling sites were identical or adjacent. Thus, intrinsic factors are the primary factors underlying the different population distribution patterns.

First, the two species belong to different kingdoms and present different historical demographics. Differences in historical demography always have a great contribution to the different population distribution patterns. Therefore, it probably was the primary intrinsic factor.

Second, larvae are the primary dispersal vector, especially during the planktotrophic developmental stage (Lutz et al. 1986; Iwasaki et al. 2006; Thaler et al. 2014). Munidopsids appear to produce very large eggs and brooded larvae with yolk sacs, consistent with lecithotrophy, and possibly a limited dispersal capability compared to the planktotrophic larvae with high dispersal capability for the bathymodioline mussels (Won et al. 2003; Miyazaki et al. 2004). At the scale of 1000 km, one could therefore expect to see differentiation between the seep and vent sites for *S. crosnieri*, as has been shown at a similar scale for the tubeworm Riftia pachyptila in the East Pacific (Coykendall et al. 2011).

Finally, the levels of genetic differentiation were also likely to be related to physiology. Miyazaki et al. suggested high adaptability of these Bathymodiolus species to deep‐sea chemosynthetic environments (Miyazaki et al. 2004). Our analysis also showed that *B. platifrons* had high adaptability to different habitats. Therefore, a possible explanation was that the higher degree of tolerance to environmental heterogeneity of *B. platifrons*, in comparison to *S. crosnieri*, should be responsible for their different population distribution patterns. However, this proposed explanation requires further proof of physiology tests.

This study provides the first documentation of the different population genetic structure and distribution patterns for *S. crosnieri* and *B. platifrons* inhabiting both deep‐sea vents and cold seep. Nevertheless, caveats are necessary with respect to the limited sample locations and small sample sizes. More sample sites and molecular markers, especially nuclear loci, should be employed in further research.

## Conflict of Interest

None declared.

## Supporting information


**Table S1.** PCR primers used in the present study.
**Table S2**. Gene flow (*N*_m_: below the diagonal) was evaluated from the values of GammaSt and *F*_ST_ for the *Shinkaia crosnieri* and *Bathymodiolus platifrons* populations.
