# Supplementary material for: Comparative population structure of two dominant species, Shinkaia crosnieri (Munidopsidae: Shinkaia) and Bathymodiolus platifrons (Mytilidae: Bathymodiolus), inhabiting both deep‐sea vent and cold seep inferred from mitochondrial multi‐genes
Source: Ecol Evol. 2016 Apr 23;6(11):3571–82. doi: 10.1002/ece3.2132 (PMC5513293; doi:10.1002/ece3.2132)
Supplement: Supplementary file 1 — Table S1. PCR primers used in the present study. Table S2. Gene flow (N m: below the diagonal) was evaluated from the values of GammaSt and F ST for the Shinkaia crosnieri and Bathymodiolus platifrons populations. [file ECE3-6-3571-s001.docx]

**Table S1.** PCR primers used in the present study.

| Gene | *Shinkaia crosnieri* | *Bathymodiolus platifrons* | Reference |
| --- | --- | --- | --- |
| COI | LCO1490 (5′-GGTCAACAAATCATAAAGATATTGG-3′)  HCO2198(5′-TAAACTTCAGGGTGACCAAAAAATCA-3′  CrustDF1(5′-GGTCWACAAAYCATAAAGAYATTGG-3′)  CrustDR1(5′-TAAACYTCAGGRTGACCRAARAAYCA-3′) | LCO1490 (5′-GGTCAACAAATCATAAAGATATTGG-3′)  HCO2198(5′-TAAACTTCAGGGTGACCAAAAAATCA-3′  CrustDF1(5′-GGTCWACAAAYCATAAAGAYATTGG-3′)  CrustDR1(5′-TAAACYTCAGGRTGACCRAARAAYCA-3′) | ([Folmer, Black et al. 1994](#_ENREF_2))  ([Radulovici, Sainte-Marie et al. 2009](#_ENREF_6)) |
| Cytb | Cytb151F (5’-TGTGGRGCNACYGTWATYACTAA-3’)  Cytb270R (5’-AANAGGAARTAYCAYTCNGGYTG-3’) | CobF (5′-GGWTAYGTWYTWCCWTGRGGWCARAT-3′)  CobR(5′-GCRTAWGCRAAWARRAARTAYCAYTCWGG-3′) | ([Merritt, Shi et al. 1998](#_ENREF_3))  ([Passamonti 2007](#_ENREF_5)) |
| 16S | 16S1472 (5'-AGATAGAAACCAACCTGG-3')  16Sbraz (5'-GACCGTGCKAAGGTAGCATAATC-3') | 16SLRJ (5'-CTCCGGTTTGAACTCAGATC-3')  16SA(5'-GGARGTASGCCCTGCCCWATGC-3') | ([Palumbi S;Martin A;Romano S 1991](#_ENREF_4)), ([Baco-Taylor 2002](#_ENREF_1)) |

**Table S2**. Gene flow (N_m_: below the diagonal) was evaluated from the values of GammaSt and Fst for the *Shinkaia crosnieri* and *Bathymodiolus platifrons* populations.

| Species | Population | WTS2 | | WTS1 | | | NIS | | WKC | | |
| --- | --- | --- | --- | --- | --- | --- | --- | --- | --- | --- | --- |
| *Shinkaia crosnieri* |  | GammaSt | Fst | GammaSt | Fst | | GammaSt | Fst |  | | |
|  | WTS2 |  |  |  |  | |  |  |  |  |  |
|  | WTS1 | 19.44 | 15.86 |  |  | |  |  |  |  |  |
|  | NIS | 2.13 | 2.59 | 29.93 | 43.98 | |  |  |  |  |  |
|  | WKC | 1.46 | 1.21 | 1.08 | 0.27 | | 1.41 | 1.04 |  |  |  |
| *Bathymodiolusplatifrons* | Population | NIS1 | | NIS2 | | | WKC | |  |  |  |
|  |  | GammaSt | Fst | GammaSt | | Fst |  | |  |  |  |
|  | NIS1 |  |  |  | |  |  |  |  |  |  |
|  | NIS2 | 5.06 | 5.60 |  | |  |  |  |  |  |  |
|  | WKC | 11.17 | 9.96 | 4.78 | | 2.37 |  |  |  |  |  |
